# Supplementary figures and images for: “It let me merge my love of teaching with research”: A qualitative investigation of the career pathways of biology education researchers
Source: PLoS One. 2024 Oct 17;19(10):e0312243. doi: 10.1371/journal.pone.0312243 (PMC11486405; doi:10.1371/journal.pone.0312243)

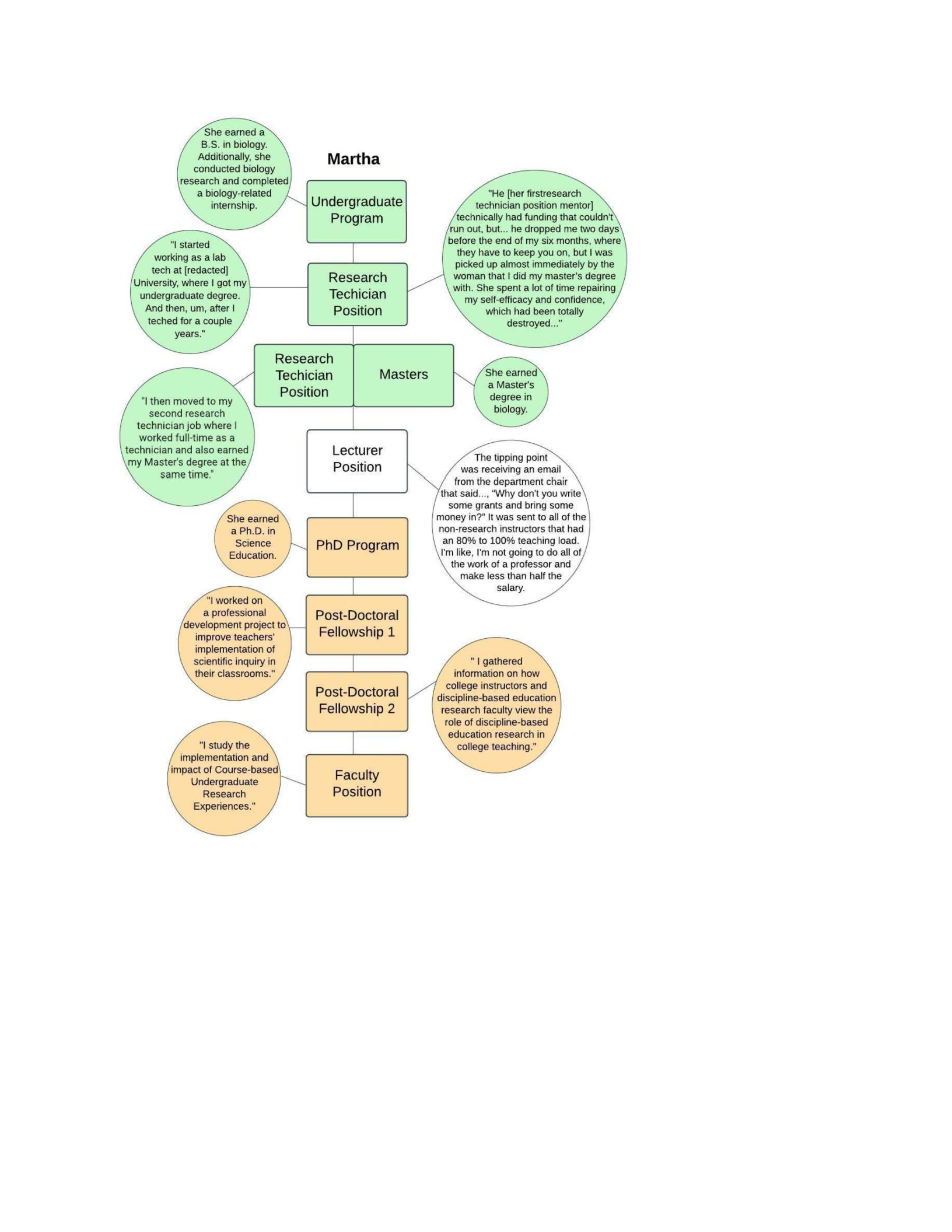

Supplement: S1 Fig — Green rectangles or circles represent a professional experience in disciplinary biology research and orange rectangles or circles represent a professional experience in biology education research. White rectangles or circles represent a professional experience where neither disciplinary biology nor biology education research was conducted. (TIF) [file pone.0312243.s003.tif]

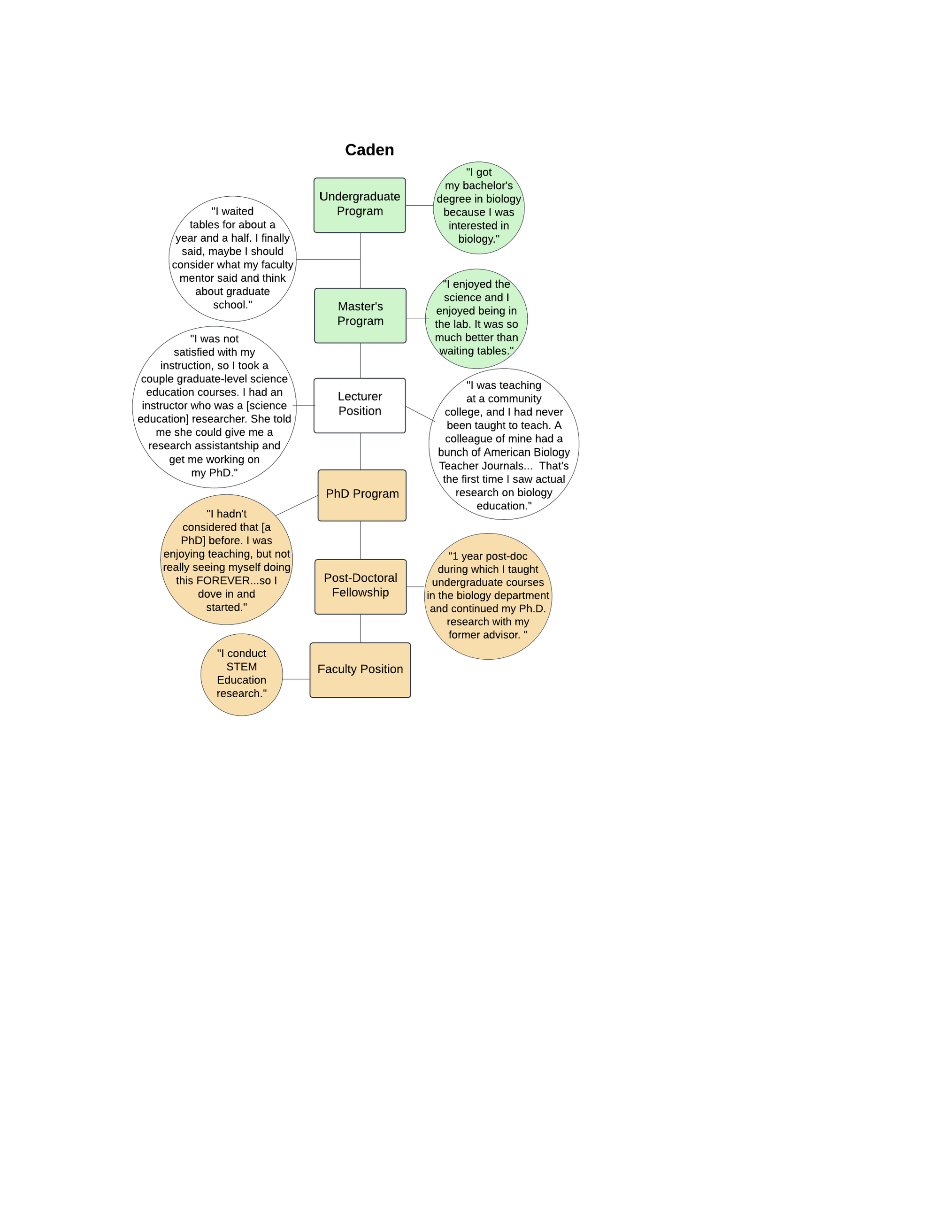

Supplement: S2 Fig — Green rectangles or circles represent a professional experience in disciplinary biology research and orange rectangles or circles represent a professional experience in biology education research. White rectangles or circles represent a professional experience where neither disciplinary biology nor biology education research was conducted. (TIF) [file pone.0312243.s004.tif]

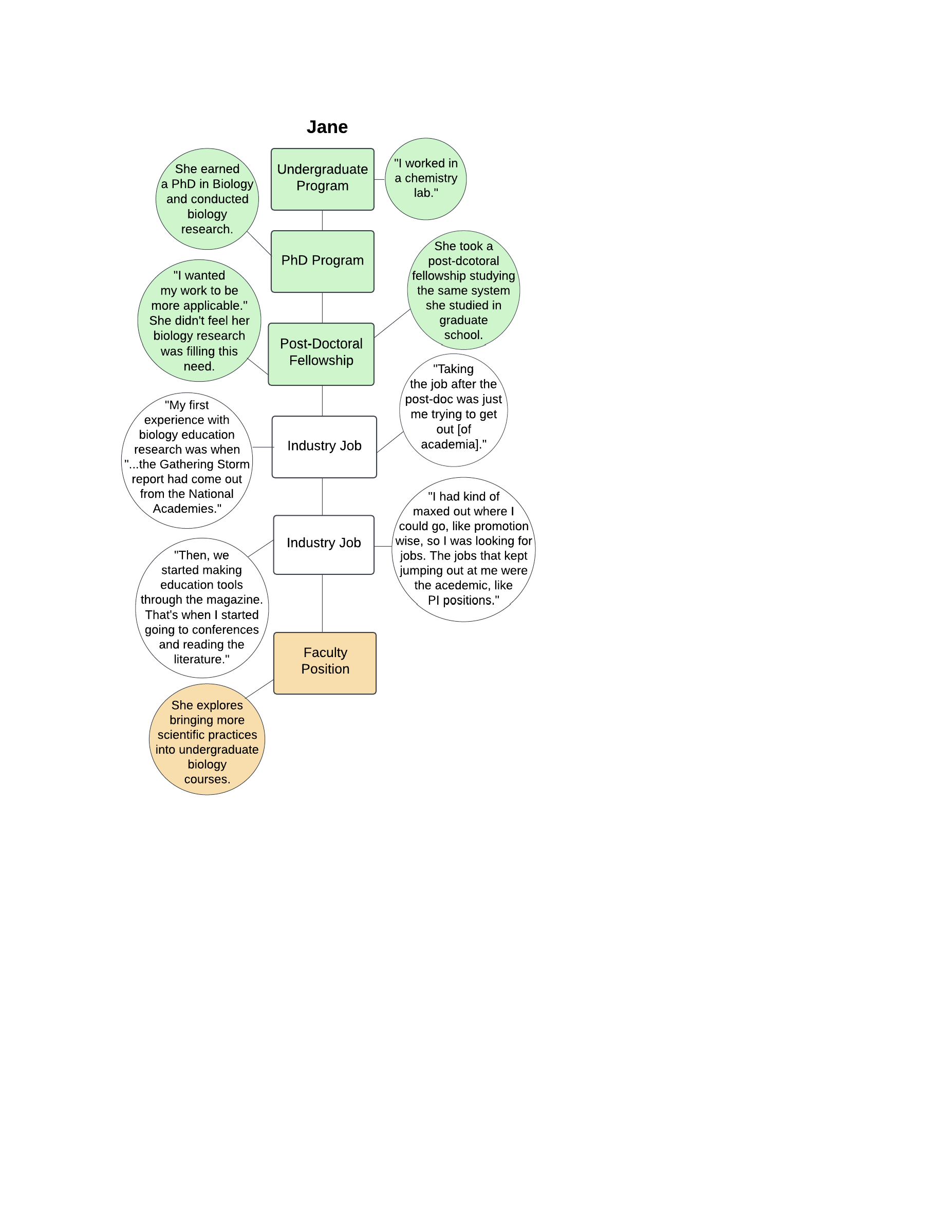

Supplement: S3 Fig — Green rectangles or circles represent a professional experience in disciplinary biology research and orange rectangles or circles represent a professional experience in biology education research. White rectangles or circles represent a professional experience where neither disciplinary biology nor biology education research was conducted. (TIF) [file pone.0312243.s005.tif]

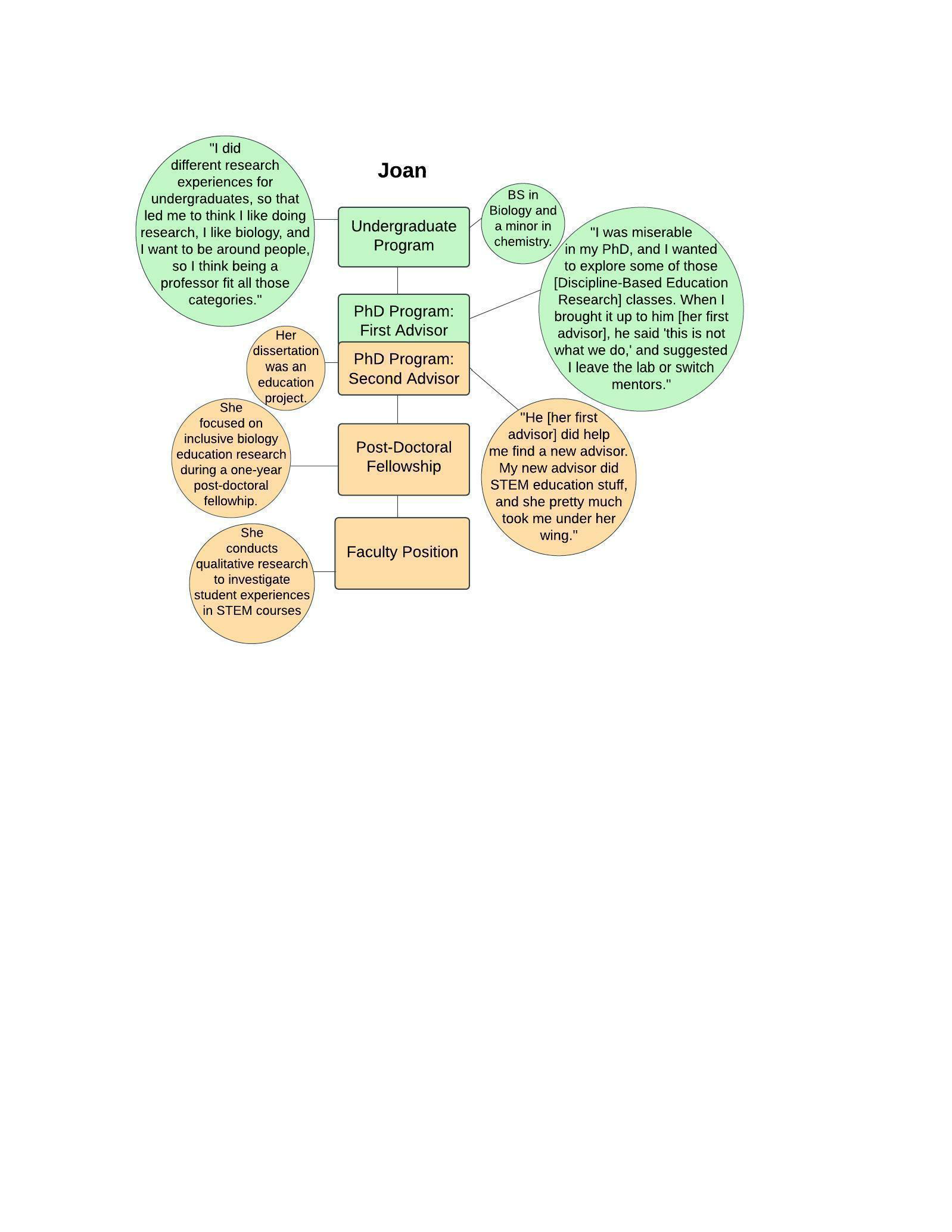

Supplement: S4 Fig — Green rectangles or circles represent a professional experience in disciplinary biology research and orange rectangles or circles represent a professional experience in biology education research. (TIF) [file pone.0312243.s006.tif]

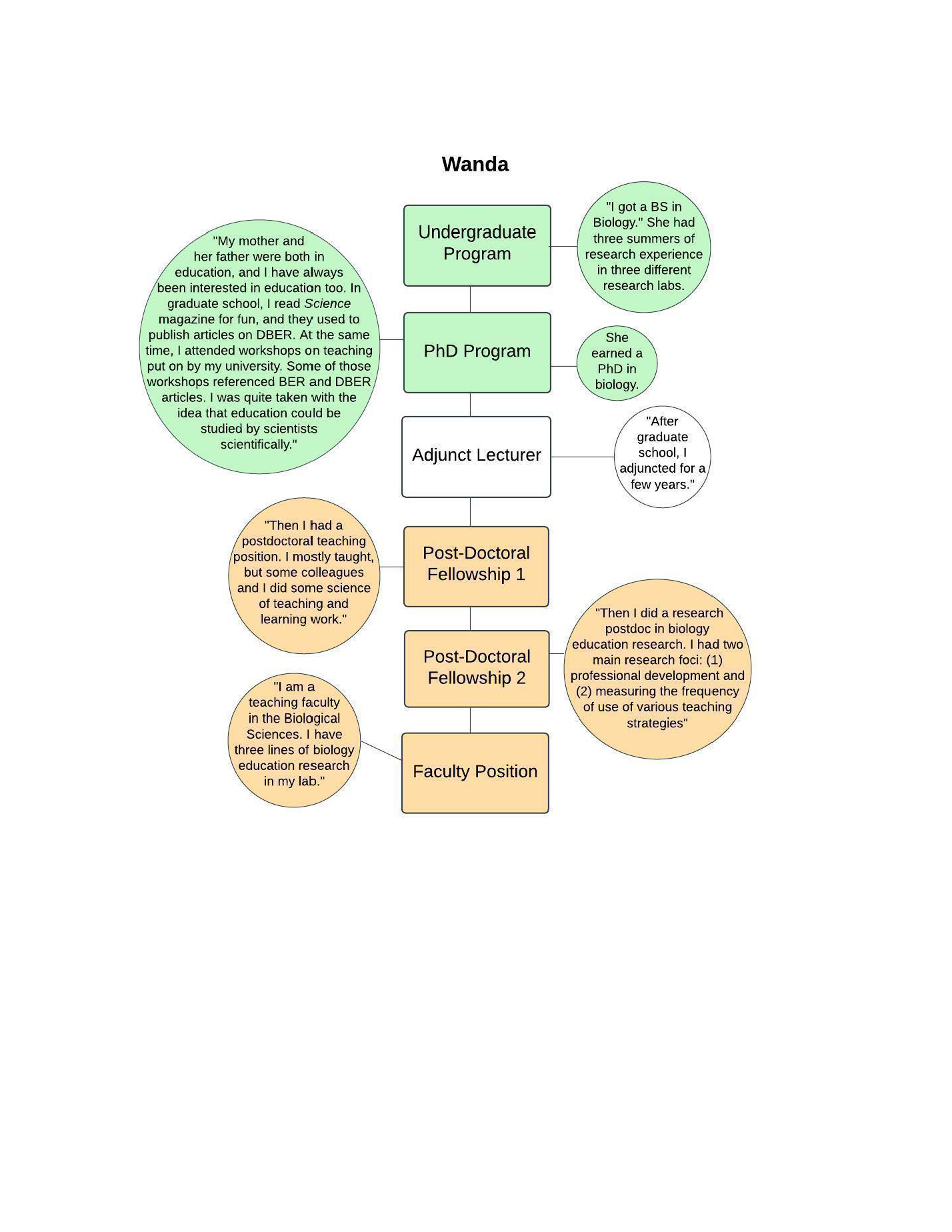

Supplement: S5 Fig — Green rectangles or circles represent a professional experience in disciplinary biology research and orange rectangles or circles represent a professional experience in biology education research. White rectangles or circles represent a professional experience where neither disciplinary biology nor biology education research was conducted. (TIF) [file pone.0312243.s007.tif]
