# Supplementary material for: “It let me merge my love of teaching with research”: A qualitative investigation of the career pathways of biology education researchers
Source: PLoS One. 2024 Oct 17;19(10):e0312243. doi: 10.1371/journal.pone.0312243 (PMC11486405; doi:10.1371/journal.pone.0312243)
Supplement: Attachment — Submitted filename: Response to Reviewers-5.docx [file pone.0312243.s008.docx]

PONE-D-24-22006

“It let me merge my love of teaching with research”: A qualitative investigation of the career pathways of biology education researchers

PLOS ONE

Dear Dr. Driessen,

Thank you for submitting your manuscript to PLOS ONE. After careful consideration, we feel that it has merit but does not fully meet PLOS ONE’s publication criteria as it currently stands. Therefore, we invite you to submit a revised version of the manuscript that addresses the points raised during the review process.

==============================

**Dear Authors, your work was reviewed and the final outcome is minor revisions. Please send the revised revision** for acceptance. Regards

==============================

Please submit your revised manuscript by Sep 07 2024 11:59PM. If you will need more time than this to complete your revisions, please reply to this message or contact the journal office at plosone@plos.org. When you're ready to submit your revision, log on to <https://www.editorialmanager.com/pone/>and select the 'Submissions Needing Revision' folder to locate your manuscript file.

Please include the following items when submitting your revised manuscript:

- A rebuttal letter that responds to each point raised by the academic editor and reviewer(s). You should upload this letter as a separate file labeled 'Response to Reviewers'.
- A marked-up copy of your manuscript that highlights changes made to the original version. You should upload this as a separate file labeled 'Revised Manuscript with Track Changes'.
- An unmarked version of your revised paper without tracked changes. You should upload this as a separate file labeled 'Manuscript'.

If you would like to make changes to your financial disclosure, please include your updated statement in your cover letter. Guidelines for resubmitting your figure files are available below the reviewer comments at the end of this letter.

If applicable, we recommend that you deposit your laboratory protocols in [protocols.io](http://protocols.io/) to enhance the reproducibility of your results. Protocols.io assigns your protocol its own identifier (DOI) so that it can be cited independently in the future. For instructions see: <https://journals.plos.org/plosone/s/submission-guidelines#loc-laboratory-protocols>. Additionally, PLOS ONE offers an option for publishing peer-reviewed Lab Protocol articles, which describe protocols hosted on [protocols.io](http://protocols.io/). Read more information on sharing protocols at <https://plos.org/protocols?utm_medium=editorial-email&utm_source=authorletters&utm_campaign=protocols>.

We look forward to receiving your revised manuscript.

Kind regards,

Yolanda Malele-Kolisa, BDS, MPH, MDent, PhD
Academic Editor
PLOS ONE

Journal Requirements:

**When submitting your revision, we need you to address these additional requirements.**

1. Please ensure that your manuscript meets PLOS ONE's style requirements, including those for file naming. The PLOS ONE style templates can be found at
   <https://journals.plos.org/plosone/s/file?id=wjVg/PLOSOne_formatting_sample_main_body.pdf> and
   <https://journals.plos.org/plosone/s/file?id=ba62/PLOSOne_formatting_sample_title_authors_affiliations.pdf>

*We reformatted our manuscript to meet PLOS ONE’s style requirements, including those for file naming.

2. Thank you for stating the following financial disclosure: "We acknowledge the National Science Foundation for providing funding to CJB (NSF-DUE-2011995)."
   Please state what role the funders took in the study. If the funders had no role, please state: ""The funders had no role in study design, data collection and analysis, decision to publish, or preparation of the manuscript.""
   If this statement is not correct you must amend it as needed.
   Please include this amended Role of Funder statement in your cover letter; we will change the online submission form on your behalf.

*We added the requested change to our cover letter in the second to last paragraph.

3. Please expand the acronym “NSF_DUE” (as indicated in your financial disclosure) so that it states the name of your funders in full.
   This information should be included in your cover letter; we will change the online submission form on your behalf.

*We added the requested change to our cover letter in the second to last paragraph.

4. We note that you have indicated that there are restrictions to data sharing for this study. For studies involving human research participant data or other sensitive data, we encourage authors to share de-identified or anonymized data. However, when data cannot be publicly shared for ethical reasons, we allow authors to make their data sets available upon request. For information on unacceptable data access restrictions, please see <http://journals.plos.org/plosone/s/data-availability#loc-unacceptable-data-access-restrictions>.

*The datasets generated and analyzed during the current study are not publicly available due to concerns of potential participant identification. Data are available on request to the corresponding author only. We are not allowed to send the interview transcripts and survey results to a third party due to limitations with our institutional review board approval (Auburn IRB protocol no. 21-354 EX 2108).

**Before we proceed with your manuscript, please address the following prompts:**

A. If there are ethical or legal restrictions on sharing a de-identified data set, please explain them in detail (e.g., data contain potentially identifying or sensitive patient information, data are owned by a third-party organization, etc.) and who has imposed them (e.g., a Research Ethics Committee or Institutional Review Board, etc.). Please also provide contact information for a data access committee, ethics committee, or other institutional body to which data requests may be sent.

*Due to the potentially identifiable information in our interview data we are unable to share a de-identified data set. The following is the contact information for Auburn University Human Research Protection Program. Email: IRBAdmin@auburn.edu; Phone: 334-844-5966.

B. If there are no restrictions, please upload the minimal anonymized data set necessary to replicate your study findings to a stable, public repository and provide us with the relevant URLs, DOIs, or accession numbers. Please see <http://www.bmj.com/content/340/bmj.c181.long> for guidelines on how to de-identify and prepare clinical data for publication. For a list of recommended repositories, please see <https://journals.plos.org/plosone/s/recommended-repositories>. You also have the option of uploading the data as Supporting Information files, but we would recommend depositing data directly to a data repository if possible. Please update your Data Availability statement in the submission form accordingly.

*As mentioned above, we are unable to share a de-identified data set due to potentially identifiable information in our interview data.

C. Please ensure that you include a title page within your main document. You should list all authors and all affiliations as per our author instructions and clearly indicate the corresponding author.

*We added a title page and - in it - included the requested information.

D. Please review your reference list to ensure that it is complete and correct. If you have cited papers that have been retracted, please include the rationale for doing so in the manuscript text, or remove these references and replace them with relevant current references. Any changes to the reference list should be mentioned in the rebuttal letter that accompanies your revised manuscript. If you need to cite a retracted article, indicate the article’s retracted status in the References list and also include a citation and full reference for the retraction notice.

*We have thoroughly reviewed our reference list to ensure it is complete and correct. We have not cited any papers that have been retracted. To respond to some reviewer comments, we have added references. We disclose that clearly in our response to reviewers. Additionally, to meet PLOS One’s formatting requirements, we reformatted our references using the Vancouver citation style.

[Note: HTML markup is below. Please do not edit.]

Reviewers' comments:

Reviewer's Responses to Questions

**Comments to the Author**

1. Is the manuscript technically sound, and do the data support the conclusions?

The manuscript must describe a technically sound piece of scientific research with data that supports the conclusions. Experiments must have been conducted rigorously, with appropriate controls, replication, and sample sizes. The conclusions must be drawn appropriately based on the data presented.

Reviewer #1: Yes

Reviewer #2: Yes

2. Has the statistical analysis been performed appropriately and rigorously?

Reviewer #1: Yes

Reviewer #2: Yes

3. Have the authors made all data underlying the findings in their manuscript fully available?

The [PLOS Data policy](http://www.plosone.org/static/policies.action#sharing) requires authors to make all data underlying the findings described in their manuscript fully available without restriction, with rare exception (please refer to the Data Availability Statement in the manuscript PDF file). The data should be provided as part of the manuscript or its supporting information, or deposited to a public repository. For example, in addition to summary statistics, the data points behind means, medians and variance measures should be available. If there are restrictions on publicly sharing data—e.g. participant privacy or use of data from a third party—those must be specified.

Reviewer #1: Yes

Reviewer #2: Yes

4. Is the manuscript presented in an intelligible fashion and written in standard English?

PLOS ONE does not copyedit accepted manuscripts, so the language in submitted articles must be clear, correct, and unambiguous. Any typographical or grammatical errors should be corrected at revision, so please note any specific errors here.

Reviewer #1: Yes

Reviewer #2: Yes

5. Review Comments to the Author

Please use the space provided to explain your answers to the questions above. You may also include additional comments for the author, including concerns about dual publication, research ethics, or publication ethics. (Please upload your review as an attachment if it exceeds 20,000 characters)

**Reviewer #1:**

**Summary**
This study surveyed and interviewed discipline-based education researchers about their career trajectories. The values and interests, role of mentorship, and factors influencing their career trajectories provide valuable insight into DBER careers. The research team used the Social Influence Model and Social Cognitive Career Theory to develop a survey and interview questions to guide their work. The work draws from two frameworks and uses them to advance knowledge of the career influences of DBER scholars. The values and interests identified and contextual factors help support current and future DBER scholars.

The survey instrument was revised based on expert feedback, and the research team focused on establishing trustworthiness throughout. Thus, the researchers carefully interpreted survey data and invited members for follow up interviews. The methods used by the research team not only ensured the privacy and consent of participants, but also were designed helped elicit honest responses.
*We appreciate the reviewer’s thoughtful summary of our work, and we address all of their comments and suggestions below.

Lines 230-234: Did patterns or themes emerge from the research described by survey responses and their open-ended career trajectory responses?

*To address this comment, we added clarifying text to better explain how we assessed participant responses. It was not part of our research design to analyze the data for patterns or themes. For example, we did not use thematic analysis. Rather, it was our design to see how the constructs from the Social Cognitive Career Theory and the Social Influence Model (i.e., values, interests, and contextual factors) played out in these participants' career paths and decision-making. With this in mind, we focused on coding the data, which represent small units of information that represent values, interests, or contextual factors. Upon reading the reviewer’s comment we re-read our paper and noticed we used the word themes in our figure to talk about the values, interests, and contextual factors, and we now call these “constructs” both in the figure and in the text, so the word theme does not appear in our paper. Additionally this reviewer comment made us realize we needed to more accurately describe our coding process. This involved removing the Hsieh & Shannon (2005) citation and replacing it with Saldana’s (2015) description of first and second cycle qualitative coding. We now describe how we used in vivo coding for our first cycle of coding and focused coding for our second cycle coding, both for the survey analysis and the interview analysis (Lines 306-397).

Lines 450-478: In addition to the Gibbs and Griffin 2013 results, are there more recent survey findings from the NIH and National Academies? It would be useful to contrast the 2013 results with opinions during/after the pandemic.

*After reviewing the literature to respond to this comment, we found several relevant data sources, but none were exactly what we thought the reviewer was asking for nor did many of them address the code “making a difference” **—** aside from McGee & Bentley (2017) **—** which is where we discussed the Gibbs and Griffin (2013) study. With this in mind, we added text and the one relevant reference we found (McGee & Bentley, 2017) (Lines 504-510; 1320-1321). Additionally, we added text and references in the considerations section concerning the impacts of COVID on STEM undergraduate and graduate students and that we do not yet know how these have or have not (likely have) impacted their career paths (Lines 1111 - 1121; 1419-1424).

New References added:

Driessen, E., Beatty, A., Stokes, A., Wood, S., & Ballen, C. (2020). Learning principles of evolution during a crisis: An exploratory analysis of student barriers one week and one month into the COVID‐19 pandemic. *Ecology and evolution*, *10*(22), 12431-12436.

McGee, E., & Bentley, L. (2017). The equity ethic: Black and Latinx college students reengineering their STEM careers toward justice. *American Journal of Education*, *124*(1), 1-36.

Sifri, R. J., McLoughlin, E. A., Fors, B. P., & Salehi, S. (2022). Differential impact of the COVID-19 pandemic on female graduate students and postdocs in the chemical sciences. *Journal of chemical education*, *99*(10), 3461-3470.

Line 1115: This could be an opportunity to change the role of professional societies and networks (SABER, PULSE, ASCB) to share educational research training and career paths with their members to reflect and improve.

*We totally agree! We added this thought to our considerations section as suggested (Lines 1146 - 1154).

**Reviewer #2:**

This is very important research in an under-researched area. Understanding the values, personal interests, and contextual factors that influence career paths in biology education research can help improve the field.

**The authors must address the following:**

The study design was not stated.

**To address this comment, we added text to the manuscript to identify our study design more clearly (Lines 132 - 134). Our study is an exploratory qualitative study in which we used qualitative methods (i.e., interviews and open-ended survey questions) to learn about the career motivations of people who pursued biology education research (i.e., biology education research faculty).

It is unclear if this is a mixed methods study; the authors mention open-ended surveys and follow-up interviews.

*To address this comment, we added text to the manuscript to identify our study design more clearly (Lines 132 - 134). Now it is clear that this is not a mixed-methods study. As stated above, our study is an exploratory qualitative study in which we used qualitative methods (i.e., interviews and open-ended survey questions) to learn about the career motivations of people who pursued biology education research (i.e., biology education research faculty).

While the recruitment methods are mentioned, the sampling technique is not clear.

*We add our sampling methods to the text, as well as the Whitehead and Whitehead (2020) citation, to address the reviewer’s comment (Lines 223 - 240; 1307-1308).

Reference added:

Whitehead, D. & Whitehead, L. (2020). Data collection and sampling in qualitative research. Nursing and Midwifery Research Methods and Appraisal for Evidence-Based Practice. 6th Edition. Sydney; Elsevier, 118-135.

It needs clarification whether the authors mean they did not collect any demographic information for participants when they say, “elected to omit demographic information on these participants to decrease chances of participant identification.” Are there any other participant characteristics collected to describe the study sample?

*We collected race and gender identity demographic information on the participants in the online survey. Specifically, we asked “What term best describes your gender identity (select all that apply)?” and “What is your race* (select all that apply). The biology education research community is growing, but the number of people who are faculty members that conduct biology education research is small, so we do not feel comfortable providing both a career timeline for each interviewed participant and their gender identity/racial information. For example, providing a participant’s career timeline, their gender identity as nonbinary, and the race information as Black would be highly identifying (this is hypothetical, but not too far away from an actual participant's demographics). Additionally, we do not provide summaries of demographic data, because some of the demographics would only have 1 participant in that category, and that is additionally problematic and identifying. Our study design does not allow us to make generalizations as it is - as we acknowledge (Lines 147-150; 199-202), so - while demographics certainly can - and likely did - impact these participants’ experiences and career trajectories, we do not directly provide this information, which we have now expanded on (Lines 220 -222). However, we do have a results subsection on sexism (Lines 924-988)because we noticed participant answers to one of the survey questions (which was also an interview question; Do you feel your gender and/or race/ethnicity had anything to do with your decision to leave a disciplinary science and enter biology education research? Why or why not?; Supplemental Materials) demonstrated they faced sexist experiences. However, only one person mentioned their race/ethnicity in response to this question. We did not include this result in our manuscript because it was too identifying. We now add this information in a paragraph in the subsection about sexism (Lines 982 - 988) and as a consideration in the considerations section of the paper (Lines 1087 - 1097). Collectively, we now acknowledge what demographic information we did collect, that we do not present it to avoid identification, and how our research decisions may have impacted our findings. To support these changes we added the following references (Lines 1408-1418):

Malone, Kareen Ror, and Gilda Barabino. 2009. “Narrations of Race in STEM Research Settings: Identity Formation and Its Discontents.” *Science Education* 93 (3): 485–510.

Marin‐Spiotta, E., Diaz‐Vallejo, E. J., Barnes, R. T., Mattheis, A., Schneider, B., Berhe, A. A., ... & Magley, V. (2023). Exclusionary behaviors reinforce historical biases and contribute to loss of talent in the Earth sciences. *Earth's Future*, *11*(3), e2022EF002912.

Robinson, W. H., McGee, E. O., Bentley, L. C., Houston, S. L., & Botchway, P. K. (2016). Addressing negative racial and gendered experiences that discourage academic careers in engineering. *Computing in Science & Engineering*, *18*(2), 29-39.

Stockard, J., Rohlfing, C. M., & Richmond, G. L. (2021). Equity for women and underrepresented minorities in STEM: Graduate experiences and career plans in chemistry. *Proceedings of the National Academy of Sciences*, *118*(4), e2020508118.

Who conducted the interviews, and what are their credentials and experience?

*We added this information in the methods section (Lines 284 - 292). We also moved our positionality statement to the beginning of the methods section and added a few relevant details about each person’s position at the time of the study, so the reader has information on each of the researcher’s background before reading the information on who conducted the interviews (Lines 152 - 184).

The authors did not mention obtaining consent for recording the Zoom interviews.

*We obtained consent from interview participants to record their interviews. This was previously mentioned in the text. However, we now added the word “ Zoom recorded” to improve clarity (Lines 210 - 217). This information is under the “Participant” subsection of the methods, which we have now renamed “Participant Recruitment” to be more clear (Line 185). Additionally, we added the fact that we obtained consent for the recorded interviews in the interview subsection of the methods as well (Lines 281-283).

Data saturation was not addressed.

*To address the reviewer’s comment, we added text and a reference to indicate that data saturation was not something we tried to nor claim to have reached (Lines 236 - 240; 1309-1310). Given the nature of our research focus (i.e., the experiences and career paths of biology education research faculty), it is likely that there are biology education research faculty that have different career paths than any of the participants in this study because each person’s exact experience and pathway is unique. With this in mind, we did not design our experiment to reach data saturation.

Data saturation makes an assumption that the population being sampled is homogenous in some way (i.e., the same themes or codes will come up repeatedly, signaling data saturation) (Tight, 2023). See below excerpt from Tight (2023):

Simply applying [Hennink and Kaiser’s (2022)](https://journals.sagepub.com/doi/10.1177/10778004231183948#bibr19-10778004231183948) guidelines to any qualitative research project, particularly when the population being sampled is not homogeneous and the subject of study is broad and/or ill-defined, is not, therefore, a sensible or defensible strategy. As [Sim et al. (2018)](https://journals.sagepub.com/doi/10.1177/10778004231183948#bibr32-10778004231183948) conclude, “defining sample size a priori is inherently problematic in the case of inductive, exploratory research, which, by definition, looks to explore phenomena in relation to which we cannot identify the key themes in advance” (p. 630).

We may agree with [Bowen (2008)](https://journals.sagepub.com/doi/10.1177/10778004231183948#bibr1-10778004231183948) that “claims of saturation should always be supported by an explanation of how saturation was achieved and substantiated by clear evidence of its occurrence” (p. 150), but have to ask how can we know whether saturation has been reached, especially if the topic being studied is complex? There is always the possibility that the next interviewee or the next focus group—particularly if we recruit outside of our comfort zone—will provide responses to challenge our existing analysis. And those responses might be just the ones we need to progress the understanding of our field further.

To recruit participants, we sent out a SABER listserv to recruit interested and consenting participants and collect survey information. Then, of those participants who consented additionally to an interview, we contacted 9 of them to gather more detailed information. We decided on this number based on convenience, which we now acknowledge in the manuscript (i.e., convenience sampling; Lines 223 - 236). However, there were common motivations, values, and interests among participants which could point towards “data saturation;” we presented these overlapping constructs in the results section.

Reference added:

Tight, M. (2023). Saturation: An Overworked and Misunderstood Concept? *Qualitative Inquiry*, *0*(0). <https://doi.org/10.1177/10778004231183948>

6. PLOS authors have the option to publish the peer review history of their article ([what does this mean?](https://journals.plos.org/plosone/s/editorial-and-peer-review-process#loc-peer-review-history)). If published, this will include your full peer review and any attached files.


If you choose “no”, your identity will remain anonymous but your review may still be made public.


**Do you want your identity to be public for this peer review?** For information about this choice, including consent withdrawal, please see our [Privacy Policy](https://www.plos.org/privacy-policy).

Reviewer #1: **Yes:** Carlos C. Goller

Reviewer #2: No

[NOTE: If reviewer comments were submitted as an attachment file, they will be attached to this email and accessible via the submission site. Please log into your account, locate the manuscript record, and check for the action link "View Attachments". If this link does not appear, there are no attachment files.]

While revising your submission, please upload your figure files to the Preflight Analysis and Conversion Engine (PACE) digital diagnostic tool, <https://pacev2.apexcovantage.com/>. PACE helps ensure that figures meet PLOS requirements. To use PACE, you must first register as a user. Registration is free. Then, login and navigate to the UPLOAD tab, where you will find detailed instructions on how to use the tool. If you encounter any issues or have any questions when using PACE, please email PLOS at figures@plos.org. Please note that Supporting Information files do not need this step.
